# Molecular Identification of *Zantedeschia* Culture with Determination of Its Morphometric and Metabolic Activities for Mediterranean Acclimatization

**DOI:** 10.3390/plants11172311

**Published:** 2022-09-03

**Authors:** Eman Tawfik, Mohamed Fathy Ahmed, Doha A. Albalawi, Bandar S. Aljuaid, Doaa Bahaa Eldin Darwish, Samy F. Mahmoud, Karim M. Hassan, Mohamed F. M. Ibrahim, Ashraf Bakry Abdel Razik

**Affiliations:** 1 Botany and Microbiology Department, Faculty of Science, Helwan University, Cairo 11566, Egypt; 2 Horticulture Department, Faculty of Agriculture, Ain Shams University, Cairo 11566, Egypt; 3 Department of Biology, Faculty of Science, University of Tabuk, Tabuk 71491, Saudi Arabia; 4 Department of Biotechnology, College of Science, Taif University, P.O. Box 11099, Taif 21944, Saudi Arabia; 5 Botany Department, Faculty of Science, Mansoura University, Mansoura 35511, Egypt; 6 Biology Department, Faculty of Science, Tabuk University, Tabuk 71491, Saudi Arabia; 7 Department of Agricultural Botany, Faculty of Agriculture, Ain Shams University, Cairo 11566, Egypt; 8 Genetics Department, Faculty of Agriculture, Ain Shams University, Cairo 11566, Egypt

**Keywords:** *Zantedeschia albomaculata*, in vitro culture, pigmentation, morphological description, anatomy, phenolics, flavonoids, GC–MS, LC–MS, raphides, antimicrobial activity

## Abstract

Calla lily (*Zantedeschia albomaculata* (Hook.) Baill.) is an herbaceous or semi-evergreen perennial grown from rhizomes. It is commonly named “Spotted Arum”. Ribosomal RNAs (rRNAs) are found in all known organisms and are known for being functionally equivalent in all of them. A completely new in vitro culture protocol was applied to *Z. albomaculata* with two hormones, 6-Benzylaminopurine (BAP) and kinetin, to obtain full growth and multiplication. Due to their highly conserved sequences, the analysis of small-subunit rRNAs (16S–18S rRNAs) can provide precise statistical evaluation of a wide variety of phylogenetic connections. As a result, the plant’s 18S rRNA gene allowed for identification and partial sequencing. Also, the traditional floral method and the novel application technique for identification were applied to *Z. albomaculata*. In this paper we systemically describe the structural strategies of the plant’s adaptation to the surroundings at the morphological, physiological, and anatomical scale. Most the essential oils and fatty acids found in *Z. albomaculata* are omega fatty acids, octadecenoic acid, linoleic acid, and palmitic acid. All these fatty acids have industrial, medicinal, and pharmaceutical applications. The significant findings are the spadix sheathing leaves, and the precipitation of raphides calcium oxalate. The mitotic index showing the division activity was recorded, and it was 17.4%. The antimicrobial activity of *Z. albomaculata* ethanol extract was performed via the well diffusion method. This extract has shown high activity against *Escherichia coli* and *Pseudomonas aeruginosa*, compared to its lower activity against *Bacillus cereus*. By defining these characteristics and in vitro culture conditions, we will be able to acclimatize the plant in greenhouses, and then transfer it to the open field. The findings of this work identified the general characteristics of *Zantedeschia albomaculata* as an ornamental and medicinal plant in order to acclimatize this plant for cultivation in the Mediterranean climate.

## 1. Introduction

In the family Araceae, the genus *Zantedeschia* contains eight species of herbaceous, perennial, flowering plants that are indigenous to southern Africa, from Malawi to South Africa. It is native to southern Africa in Lesotho, South Africa, and Eswatini. Except for Antarctica, the genus has been introduced to every continent. Common names for this flower include arum lily, calla lily, and white spotted lily. Species and cultivars are highly appreciated and frequently cultivated as attractive plants due to their vivid blooms and leaves [1]. All *Zantedeschia* species produce huge, showy flower spathes, and they are frequently planted for both cut flowers and aesthetic purposes. Although *Zantedeschia* are generally hardy plants, certain varieties are more winter-resistant than others. Arum lilies, which are robust outdoor plants with huge white flowers, and calla lilies, which are less hardy but have blossoms in a variety of hues, including yellow, orange, pink, and purple, as well as leaves with white spots [2].

*Zantedeschia* irritates skin on touch in a manner similar to other members of the Araceae family. Due to the presence of calcium oxalate crystals in the form of raphides, *Zantedeschia* species are also poisonous. The plant is poisonous in all parts, usually causing localized discomfort or a burning feeling in the mouth, but also occasionally causing vomiting and diarrhea. However, leaves can occasionally be prepared and consumed [3].

Internal contamination is the biggest issue, and the availability of different sources of explant material makes it difficult to establish contamination-free material in vitro [4,5,6]. Tissue culture techniques have been developed as an alternative planting material for tuber production in *Z. aethiopica* [7,8]. When rhizomes are prepared with heat and a fungicide and then given an NaOCl treatment, the traditional disinfection procedure seems to be ineffective. Low rhizome bud survival rates were another effect of this [9]. Internal impurities like germs can be extremely challenging to remove. Additionally, we were unable to locate any reports of *Z. albomaculata* being propagated in vitro.

Several studies have used 18S rRNA sequences with a primary focus on the evolution of terrestrial plants and the classification of those plants into separate phylogenetic groupings [10]. The conclusions of plant phylogenies based on 18S rRNA or related sequences can be improved by greater sampling. Due to the small amount of known genome/gene sequences covering a diverse variety of plants, this task appears to be difficult. Unfortunately, the majority of genomic data is still restricted to model plants and a small number of crops, and little is known about wild or some cultivated species with varying degrees of economic and medical value [11,12,13].

Every plant in nature must have unique morphological and anatomical traits. The plants belonging to the same genus will exhibit morphological and anatomical variations as well as different ages, or more specifically, when they are not yet mature. This phenomenon happens as a result of the young plants’ morphological and anatomical structures’ imperfectly comprehensive growth and development. Identifying morphological and anatomical traits might also be an attempt to determine which genus the plants should belong to [14].

The aim of this study was to develop a complete protocol for the in vitro propagation of *Zantedeschia albomaculata*; the comprehensive identification of its morphological, physiological, and anatomical features; and, an estimate of the mitotic index to explain the cell division activity and antimicrobial activity against some bacterial strains.

## 2. Results

### 2.1. Plan Identification 

The identification of *Z. albomaculata* was performed via three methods: floral identification for the different vegetative parts, digital identification using the mobile application LeafSnap, and finally molecular identification using 18srRNA. 

#### 2.1.1. Floral Identification 

**Figure 1:**
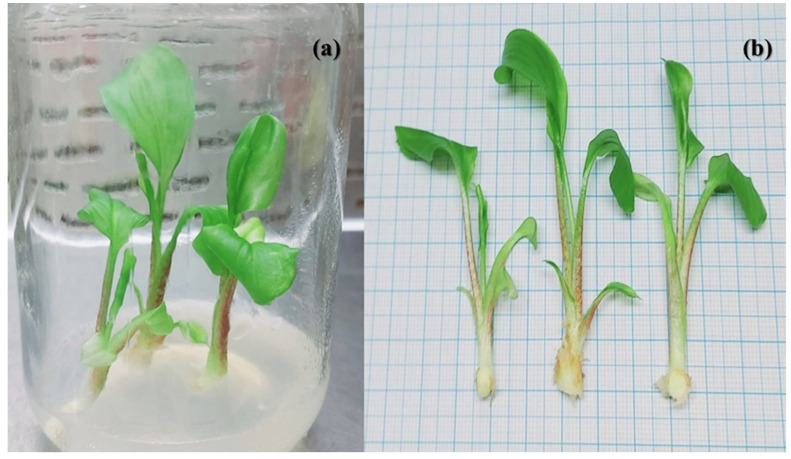
Four-week-old in vitro culture of *Zantedeschia* grown on MS media (**a**) and front look (**b**) supplemented with 3.0 BAP and 0.5 kin.

The floral identification of *Zantedeschia* is as follows: *Zantedeschia albomaculata* is an eastern species that grows in marshy soils on rocky or grassy mountainsides and is adapted to summer showers. The leaves of this medium-to-tall plant are shaped like arrows. It is a deciduous Arum with leaves that resemble arrows. The whole plantlet structure is illustrated in Figure 1. 

#### 2.1.2. The Digital Identification of Zantedeschia

**Figure 2:**
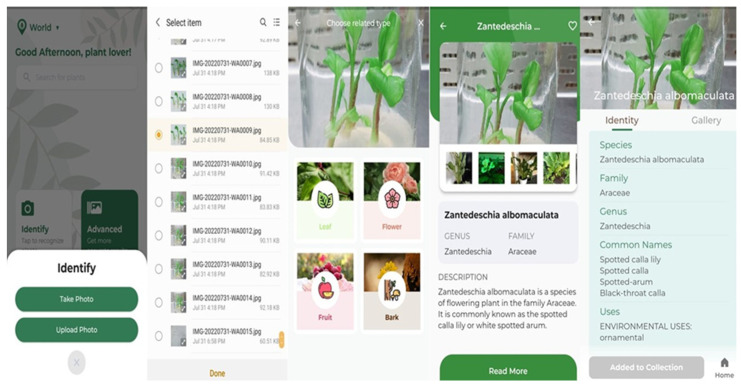
Digital identification of *Zantedeschia albomaculata* using the LeafSnap application. (The sequential images illustrate the identification steps from left to right).

This identification was performed via the “LeafSnap” mobile application, and it was identified as *Zantedeschia albomaculata*. The steps followed for identification were illustrated in Figure 2 in sequential order from left to right.

#### 2.1.3. The Molecular Identification of Zantedeschia 

**Figure 3:**
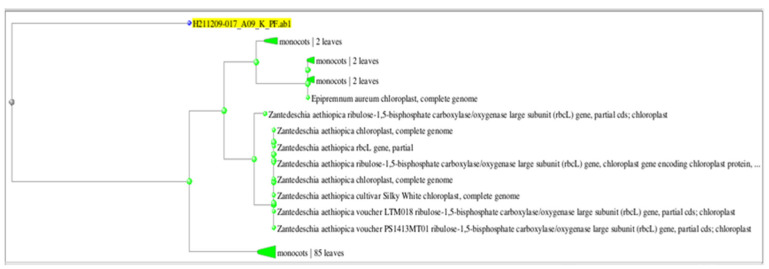
Phylogenetic tree of *Zantedeschia albomaculata* based on molecular identification.

Following DNA extraction, eukaryote-specific primers were used in the 18S rRNA-PCR technique to identify the species at the molecular level. The product was sequenced, and the resulting sequence was aligned with NCBI data to identify the plant species. The plant was identified as *Zantedeschia albomaculata* with a 92.27% identity rate (*Zantedeschia albomaculata* plastid partial rbcL gene for ribulose-biphosphate carboxylase) and accession number (AM905762.1). Figure 3 depicts the phylogenetic tree obtained from NCBI alignment for the query sequence. 

### 2.2. Morphological Description 

Variable morphological measurements were recorded for the description of *Zantedeschia* plant. The data were estimated after 4 weeks of in vitro culture on MS media and recorded in Table 1 and Figure 1. The stem was mosaic with colored dots, and the leaf is curved and bending outward and of the spathe-leaf type. The leaves are arrow-shaped, semi-erect, broad, wavy, and covered with sheath. 

### 2.3. Physiological Parameters 

#### 2.3.1. Measured Metabolites 

Some of the essential primary and secondary metabolites were estimated for *Zantedeschia albomaculata* after 4 weeks of in vitro culture. These metabolites included chlorophyll pigmentation (chlorophyll A and B), carotenoids, xanthophyll, total protein, phenolic compounds, and flavonoids (Table 2). These metabolites have essential roles in plant activity and viability. The chlorophyll index illustrates the activity of photosynthetic rate, and it was observed that the chlorophyll index for the fresh green leaves was 3.1. However, these leaves wilt rapidly and are transferred into yellow leaves within days if the incubation conditions are not suitable. The chlorophyll index for these yellow leaves decreased more rapidly and was recorded as 1.1. 

#### 2.3.2. GC–MS for Oil Content 

**Figure 4:**
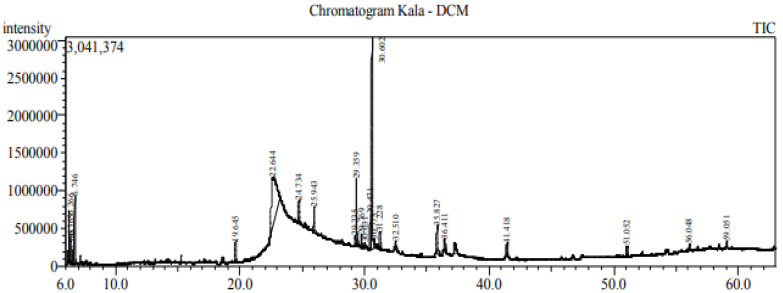
GC–MS chromatogram of the essential oil of *Zantedeschia*.

GC–MS analysis of essential oils extracted from *Zantedeschia* is represented with total ion chromatogram in Figure 4, and the results compiled in Table 3 revealed 19 compounds, representing more than 60% of the total peak area of chromatogram. The major compounds were cis-Vaccenic acid (31.75%), which is an omega-7 fatty acid; 9-Octadecenoic acid (Z)-, oxiranylmethyl ester (23.75%); Oleoyl chloride (6.89%); 9,12-Octadecadienoyl chloride, (Z,Z)- (3.65%) which is linoleic acid (polyunsaturated omega-6 fatty acid); hexadecanoic acid, trimethylsilyl ester (1.99%) (derivative of palmitic acid). These compounds and fractions were identified in the provided report sheet from the data obtained based on the MS and UV spectrum. 

So, the most essential oils and fatty acids are omega fatty acids, Octadecenoic acid, linoleic acid, and palmitic acid. Omega-6 fatty acids are a type of polyunsaturated fat that are good for the heart and appear to protect against heart disease. Omega-7 fatty acids are a class of unsaturated fatty acids and widely used in cosmetics due to their moisturizing properties. Octadecenoic acid is saturated fatty acid and used in hardening soaps, softening plastics, and in making cosmetics, candles, and plastics. Linoleic acid is polyunsaturated fatty acid and used as emollient or moisturizer for skin, nails, and hair. Palmitic acid is a saturated long-chain fatty acid used to produce cosmetics and soaps.

#### 2.3.3. LC–MS for Ethanol Extract 

**Figure 5:**
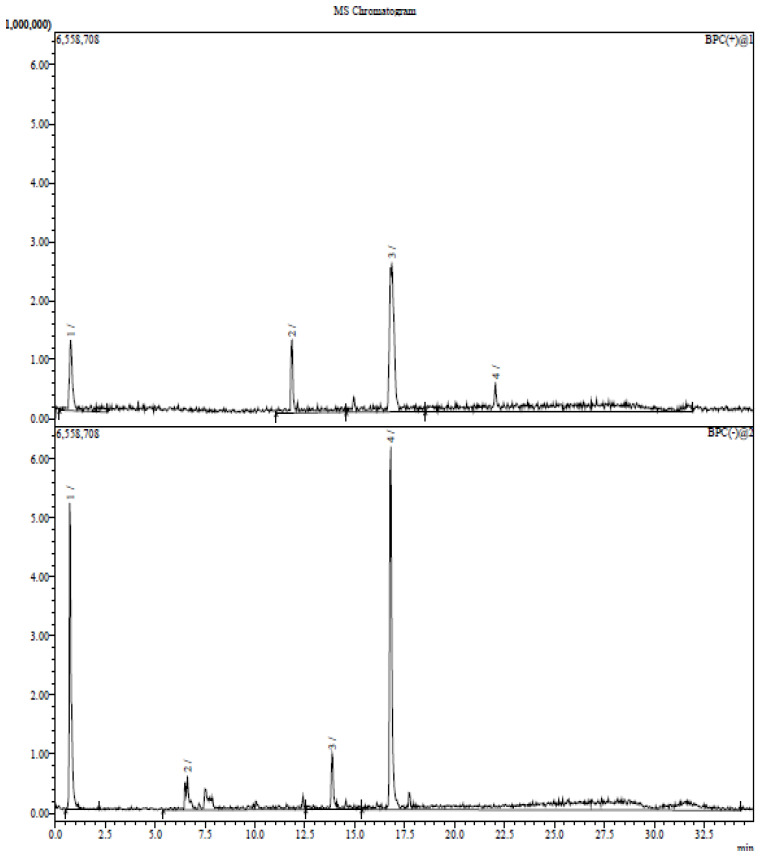
LC–MS chromatogram for the fractions of the *Zantedeschia* plant.

The total fractions (phenolic, flavonoid, and terpenoid) in the *Zantedeschia* plant were identified using LC–MS analysis. The identified fractions are tabulated in Table 4. Some of the fractions are illustrated in Figure 5. These compounds and fractions were identified in the provided report sheet based on the data obtained. 

The essential compounds obtained from LC–MS have antibiotic, antioxidant, and anticancer activities. Vanillic acid is a tannin and exhibits diverse bioactivity against cancer, diabetes, obesity, neurodegenerative, cardiovascular, and hepatic diseases by inhibition of the associated molecular pathways. Caffeoylquinic acid (CQA) is one of the phenylpropanoids which exhibit various bioactivities such as antioxidant, antibacterial, anticancer, antihistaminic. Chlorogenic Acid is a polyphenol and the ester of caffeic acid and quinic acid that has potential antioxidant activity. Roseoside is known as rose oxide, which is a class of monoterpenes used for high-value perfume and cosmetics. 

### 2.4. Anatomical Studies 

**Figure 6:**
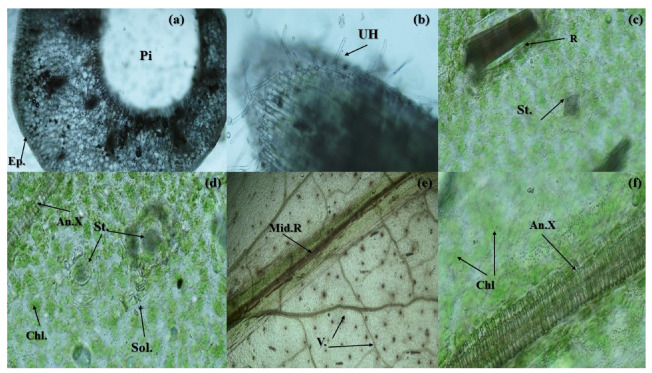
Anatomical features of *Zantedeschia*. (**a**) T.S in stem; (**b**) V.S in root; (**c**–**f**) T.S in leaf. The symbols refer to: Pi: pith, Ep.: Epidermis, UH: unicellular hair, R: raphides Ca. oxalate, St.: stomata, Chl.: chlorophyll pigmentation, An.X: annular xylem vessels, Sol.: solitary Ca. oxalate, Mid.R: mid-rib, V.: lateral veins. (BAUSCH&LOMB, 150X power).

The variable *Zantedeschia* organs have varied and qualitatively described anatomical features in the stem, root, and leaf (Figure 6). The stem is hollow with elongated epidermal cells and oil precipitation in cells. The root is very tiny and covered with unicellular hairs on surface. The leaves have obvious chlorophyll pigmentation aggregates as well as Ca. oxalate precipitation (raphides and solitary). The stomata in leaves are of paracytic type with two subsidiary cells parallel to the guard cells. 

### 2.5. Cytological Studies 

**Figure 7:**
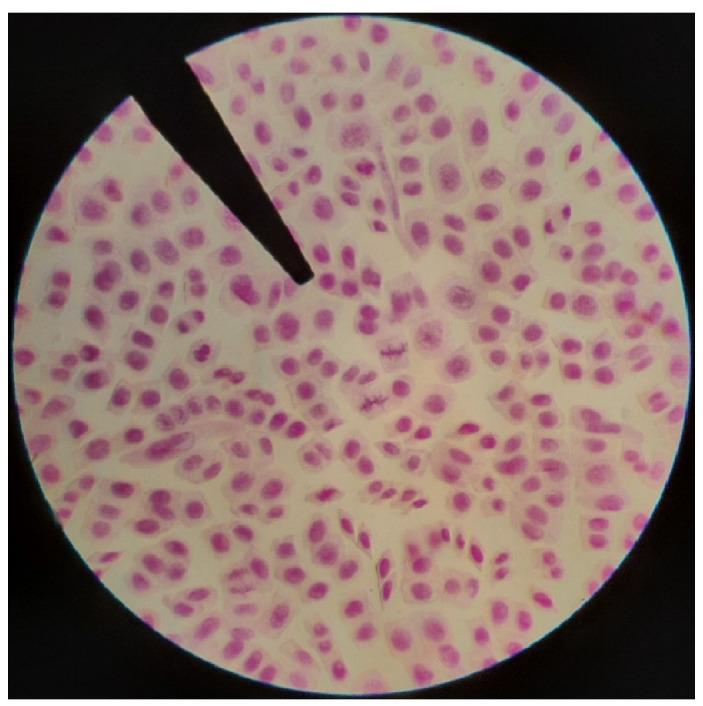
Mitotic slide from Zantedeschia root tips illustrating the dividing cells (BAUSCH&LOMB, 150X power).

The mitotic index value in root tips cells of *Zantedeschia albomoculata* was calculated according to the percentage of the dividing cells (Figure 7), and it is recorded as 17.4%. The total chromosome number of *Zantedeschia* is 2n = 32.

### 2.6. Antimicrobial Activity 

**Figure 8:**
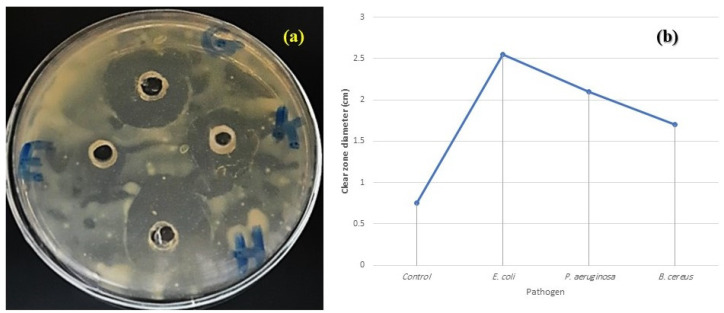
LB agar plate (**a**) showing the antimicrobial activity of *Zantedeschia* extract against (**b**) Histogram of (H): *E. coli*, (G): *P. aeruginosa*, (K): *B. cereus*, compared to control ethanol (E).

The ethanolic extracts of the Zantedeschia plant possess antimicrobial activity, as it could inhibit the growth of tested food pathogens and spoilage microorganisms. This inhibition is achieved via the clear zones (Figure 8). The extract was more resistant to *E. coli* followed by *P. aeruginosa*, and less resistant to *B. cereus*. This antimicrobial activity explained the usage of this plant as a medicinal plant. 

## 3. Discussion

This study introduced a whole protocol for the in vitro culture with a complete definition for the variable morphological, physiological, anatomical, antimicrobial, and molecular behavior of *Z. albomoculata*. In this study, the in vitro culture of *Z. albomoculata* was supplemented with 3 BAP and 0.5 Kin. The growth regulators used in the first experiment in the multiplication stage are cytokinins. This is closely related to Ruiz et al. [7] who initiated *Zantedeschia aethiopica* culture and supplemented the media with 1 mg dm-3 BAP; and Kulpa [15] who supplemented the MS medium with 1.0 BAP and 2.5 BAP. The effectiveness of BAP for the multiplication of plants of the family Aracaeae was confirmed by [16]. Chang et al. [17] modified a method for the micropropagation of *Zantedeschia albomaculata* from shoot tip culture with the application of several phytohormones (TDZ, BA, Kinetin, 2iP, IAA, NAA, and IBA). However, in this study only two growth hormones (BAP and kinetin) were used to achieve more reproducible results. Also, Tawfik and Fathy [18] proved that free-hormones MS media can be applied for the multiplication of *Gardenia jasminoides* without any growth defect. 

Morphology describes a plant’s external appearance. It is considered to be the most crucial foundation for taxonomic description. Additionally, morphology is seen as an antiquated method but is nevertheless a crucial foundation for dealing with taxonomic difficulties. The most essential indicator for identifying plants visually is the morphological structure, so studying morphology is thought to be an important approach to determine the easiest technique for researchers to clarify the genus. Consequently, the identification and classification of the diversity of plants should be considerably simpler, particularly when naming the species, family, or kingdom [19,20,21,22].

For more or less a century and a half, the features of plant internal structure have contributed to plant systematics. Additionally, phylogenetic relationships have been identified and determined using the anatomical structure of plants. Light microscopes, simple techniques, and electron microscopes are used to observe the anatomical properties. The anatomy of organs, which includes leaf, stem, root, and flower, is one of the most fundamental and taxonomic components of anatomical characteristics. Additionally, the anatomical traits can support the elaboration of a genetic relationship or phylogenetics [23].

Similar to other Araceae plants, *Zantedeschia albomoculata* has calcium oxalate crystals that are insoluble. When consumed, these crystals hurt, make swallowing challenging, temporarily make one hoarse, and induce edema. This is due to the fact that these crystals puncture the delicate tissues in the throat, gums, and tongue. Also typical is GI tract irritation. The mucilage of the cells containing raphides needles is a polysaccharide from a histochemical perspective since it colors red when subjected to Schiff’s reagent. In general, the mucilage idioblasts connected to the vascular bundles and the epidermis have been thought of as water storage cells and are therefore important for adaptation in hotter environments [24]. The number of stomata on both leaf surfaces, the lack of any compartmentalization, the fine structure of the spongy parenchyma, including the arrangement of the cells and the distribution of chloroplasts along the cell walls facing intercellular spaces, may all reduce the amount of CO_2_ diffusion resistance [25]. It has been demonstrated that plants are very adaptable to variations in spectral quality in terms of their physiology, morphology, and anatomical structure [17,26].

Numerous studies have looked at the effectiveness of plant extracts and their antimicrobial properties in preventing the growth of bacteria that cause food poisoning and spoiling. Some researchers have hypothesized that terpenoid, alkaloid, and phenolic compounds found in plant extracts act as antimicrobial agents by disrupting the microbial cell membrane and causing a flux of protons to move outward, causing cell death, or by impairing enzymes required for the biosynthesis of amino acids [27,28,29,30,31,32]. Plant extracts are regarded as readily biodegradable, nutritionally safe sources of antibacterial agents [33,34].

Many studies have proved that *Zantedeschia* species have chromosome number 2n = 32 [19,20,21]. However, no previous work has estimated the mitotic index in *Zantedeschia* to illustrate the activity and viability of cell division. 

Phytochemical and pharmacological studies have shown that compounds found in *Z. aethiopica* extracts have antifungal, antibacterial, and antioxidant properties. Both Nielsen et al. [35] and Pratush et al. [36] evaluated the antibacterial activity of methanol and ethanol extracts of the leaf and stem of *Z. aethiopica*, which exhibited activity against different bacterial strains. 

## 4. Materials and Methods

### 4.1. Plant Material

The explant of *Zantedeschia* was introduced into “Vitro Plant Labs” (plant tissue culture specialist)—Egypt, where the practical tissue culture work was performed. 

### 4.2. In Vitro Culture Conditions and Multiplication 

The protocol for the establishment of the *Z. albomaculata* culture was as follows: “segments were sterilized and cultured on free MS media for one month. The explants were defoliated and washed carefully in fluid tap water to eliminate all the stacked dust/soil practices. Tailed with surface sterilization of the explants were using 20% Clorox + 0.1% HgCl_2_ for 20 minutes and washed 4 to 5 times with double distilled sterile water. Excising and procedure of culture for explants stem nodal was performed under sterilized condition. MS basal medium contains required nutrients of macro- and micro-elements for the in vitro cultured plants as described by Murashige and Skoog [37]. The medium was allocated into incubation jars where each jar contained 50 mL. Stem nodal cultures were incubated at 25 ± 2 °C and satisfactory fluorescent light of 3000 Lux for 16-hour photoperiod provided by cool, white, fluorescent lamps”.

The shoots formed as a result of the establishment process were excised and transferred into a new multiplication MS medium supplemented with 3 mg/L of BAP + 0.5 mg/L Kin in order to obtain micro-shoots required for the multiplication experiment [15]. The multiplication process was repeated as sub-culture for three times, in order to record the study data. 

### 4.3. Plant Identification 

The identification of *Z. albomaculata* was performed via three methods: floral identification for the different vegetative parts, digital identification using the mobile application LeafSnap, and finally molecular identification using 18srRNA. 

#### 4.3.1. Floral Identification 

The Boulos [38], and Kadereit and Jeffrey’s [39] protocol was followed for the morphological identification and description of the cultured *Zantedeschia* samples.

#### 4.3.2. Digital Identification

Researchers from the Smithsonian Institution, the University of Maryland, and Columbia University are the creators of the LeafSnap series of computerized field guides. Through the use of free mobile apps, tree species can be determined from images of their leaves by using visual recognition software [40] (http://leafsnap.com/dataset/, accessed on 12 August 2022). This application is used to identify *Zantedeschia* sample via leaf. 

#### 4.3.3. Molecular Identification 

##### DNA Extraction

The whole genomic DNA was isolated according to the method of Edward et al. [41], as follows: “500 mL of CTAB buffer (2% CTAB, 2% polyvinylpyrrolidone, 1.4 M NaCl, 20 mM EDTA pH 8.0, 100 mM Tris HCl pH 8.0) was added to 0.5 g of specimen’s leaflet. The sample was heated in a water bath at 65 °C for approximately 1 h. One volume of chloroform-isoamyl alcohol (24:1) was added to the sample and mixed by inversion for 10 min and then centrifuged for 30 min at 13,200× *g*. The aqueous phase was collected into a clean microcentrifuge tube and the rest was discarded. Two volumes of absolute ethanol were added with 0.1 volumes (approximately 50 mL) of sodium acetate 3M pH 5.2 and mixed gently. The sample was left for at least 20 min at −20 °C and then centrifuged for 30 min at 13,200 g. The supernatant was discarded, and the pellet was rinsed in 70% ethanol and dried at room temperature. The pellet was dissolved in 50 µL of TE buffer (1 mM Tris HCl pH 8.0, 0.1 mM EDTA pH 8.0).” 

##### 18S rRNA PCR Amplification 

PCR amplifications of *Zantedeschia* 18S rRNA gene, from the purified genomic DNA, were carried out using the primer sets, EUK1.f, 5′-AGCGGAGGAAAAGAAACTA -’3; and EUK2.r, 5′-TACTAGAAGGTTCGATTAGTC -’3. PCR reaction mixture, 25 µL, contained 12.5 µL of master mix, 1 µL of each primer, and 50 ng DNA template. PCR was performed with an initial denaturation step of 5 min at 95 °C. The PCR reaction continued with 35 cycles of 30 sec at 94 °C, 30 sec at the annealing temperature of 54 °C, and 1 min extension at 72 °C. The final extension was at 72 °C for 10 min for one cycle. The purified 18S rRNA gene amplicons were analyzed directly by sequencing, using an ABI 3730xl DNA sequencer by using forward and reverse sequences, and combining the Traditional Sanger with the new 454 technology (GATC Company, Germany) [42].

### 4.4. Morphological Description 

The following eight morphological biometric parameters were measured for all these treatments: “plantlet fresh weight, shoot length, branch number, root number, root length, leaf length, leaves number and leaf weight”. Seven to ten replicates for each parameter were measured for accurate mean results.

### 4.5. Physiological Parameters 

#### 4.5.1. Chlorophyll Index 

Chlorophyll index is applied to calculate the total amount of chlorophyll in plant leaves. It was measured using OPTI-SCIENCES (CCM–200 plus) to indicate the photosynthesis rate in *Zantedeschia sp*. 

#### 4.5.2. Pigmentation 

Chlorophyll a, b, and carotenoids were measured in the plantlets’ fresh leaves according to the method of Metzenr et al. [43]. “In 5 mL of 85% acetone, a known weight of fresh leaves (0.5 g) was homogenized. The pigment-containing supernatant was made up to a definite volume (10 mL) with 85% acetone after centrifugation. At three wavelengths (452, 645 and 664 nm), the extract was compared to a blank of pure 85% aqueous acetone using a colorimeter. The concentration of chlorophyll a, b and carotenoids were calculated as µg/g leaf fresh weight using the following equations”:

$$\begin{array}{c} Chl.\;A=\left(\left(10.3*A664\right)-\left(0.918*A645\right)\right)\\ Chl.\;B=((19.7\;*\;A645\;(3.87\;*\;A664))\\ Carotenoids=\left(\left(4.3*A452\right)*\left(\left(0.0265*Chl.A\right)+\left(0.426*Chl.B\right)\right)\right) \end{array}$$

Then, the fractions were calculated as mg/g fresh weight:

$$Conc.=\left(\frac{Fraction*Dilution}{1000}\right)$$

Equations for the calculation of concentrations of lutein (xanthophyll pigmentation) in *Zantedeschia* leaves were obtained according to the method of Bulda et al. [44], using specific absorption coefficients of lutein at 480 and 495 nm, as follows:

$$C_{let}=\left(\left(11.51*A480\right)-\left(20.61*A495\right)\right)$$

where *A* is absorption of the solution at 480 and 495 nm, *C_let_* is the concentration of lutein as a type of xanthophyll pigmentation; concentration of lutein (mg/L).

#### 4.5.3. Total Proteins 

The total protein was extracted according to the method of Bradford [46] and some modification by Hussien [45], as follows: “Weight 0.5 g of leaves was weighted and grind well with 0.5 mL of [2x] Bradford reagent. Then vortex 10 min and centrifuge for 15 min at 24,104 g at 4 °C. The supernatant contained the total protein content of plant species”. Finally, the protein concentration was estimated according to the method of Bradford [46], as follows: “0.1 mL of supernatant was pipette into a test tube and 5 mL of protein reagent was added, mixed and measured by spectrophotometer at wavelength 595 nm. The concentration of protein was determined from the protein standard curve (using bovine serum albumin).” The concentration was calculated according to the following equation:

$$X+\left(\frac{Y-0.030}{0.007}\right)$$

where *X* was the protein concentration (mg/g), and *Y* the absorbance (nm).

#### 4.5.4. Total Phenolic Compounds

Air-dry powdered *Zantedeschia* (1 g) was extracted by stirring 30 ml methanol (80%) at room temperature until the extraction solvent became colorless. Total phenolic contents were determined according to the method described by Wang et al. [47], using Folin-Ciocalteu reagent and gallic acid as a standard. Briefly, 0.5 ml of filtered extract was added to a test tube containing 2.5 mL Folin-Ciocalteu’s reagent (diluted with ethanol 1:1), 2 mL of Na_2_CO_3_ (7.5%) and mixed well. After 15 min incubation at room temperature, the absorbance of mixtures was recorded spectrophotometrically at 765 nm using a Jenway 6405 UV-Vis spectrophotometer. The total phenolic content was calculated from a calibration curve of gallic acid standard solutions and expressed as mg gallic acid equivalent (GAE) per gram of extract (mg GAE/g dry weight of extract).

#### 4.5.5. Total Flavonoids 

The total flavonoids content of *Zantedeschia* extract was determined using aluminum chloride colorimetric assay [48]. An amount of 0.5 ml of the extract was added to 150 µL of 5% NaNO_3_ and allowed to stand for 6 minutes. Then, 150 µL of 10% AlCl_3_ solution was added and allowed to stand for 6 min, after which 200 µl solution of 1 M NaOH was added, and then the mixture was completed to 5 mL with methanol and mixed well. After incubation for 15 min, the absorbance was measured spectrophotometrically against a blank at 510 nm. The total flavonoids content was expressed in milligrams of quercetin equivalents (QE) per gram extract (mg QE/g). The standard curve of quercetin was used for the calculation of total flavonoids.

Both the total phenolic compounds and total flavonoids content were calculated according to the following equation: 

$$Concentration\frac{mg}{g}=\left(\frac{\left(R-B\right)*Dilution\;factor*Factor}{1000}\right)$$

#### 4.5.6. Total Oil extract and GC-MS Analysis for Zantedeschia Essential Oils

The total oil of *Zantedeschia* was obtained by the hydro-distillation technique using Clevenger’s apparatus. One hundred grams from plant materials were placed in a two-liters round bottom flask and distilled water was added and mixed thoroughly. The contents of the flask were boiled gently for four hours until the volatile oil was distilled. The crude volatile oil of the plant was transferred by means of a pipette into a separate brown glass bottle. Anhydrous sodium sulphate was added and agitated gently to absorb the water, and the clear oil was decanted into the brown glass bottle and kept in the refrigerator until needed for analysis [44]. After that, the oil was analyzed using GC–MS technique.

Mass spectra were recorded using a Shimadzu GCMS-QP2010 (Tokyo, Japan) equipped with Rtx-5MS fused bonded column (30 m × 0.25 mm × 0.25 µm film thickness) (Restek, Bellefonte, PA, USA) equipped with a split-spitless injector. The initial column temperature was kept at 45 °C for 2 min (isothermal) and programmed to 300 °C at a rate of 5 °C/min, and kept constant at 300 °C for 5 min (isothermal). The injector temperature was 250 °C. The helium carrier gas flow rate was 1.41 mL/min. All the mass spectra were recorded applying the following condition: (equipment current) filament emission current, 60 mA; ionization voltage, 70 eV; ion source, 200 °C. Diluted samples (1% *v*/*v*) were injected with split mode (split ratio 1:15) [49].

#### 4.5.7. Preparation of Zantedeschia Methanol Extract and LC–MS

About 10 g of *Zantedeschia* were transferred into a screw-capped extraction tube and extracted with 10 mL of 70% aqueous methanol at 75 °C for 60 min. After centrifuging at 4000 rpm for 5 min, 3 mL of supernatant was transferred to a 5 mL vial. The solution was filtrated through a syringe filter with a 0.2 μm membrane for the quantification of apigenin and apigenin 7-glucoside in the plant material by LC–MS.

### 4.6. Anatomical Description 

Traditional botanical microtome technique is used to make thin cuts for examination under a microscope. A blade is used to cut the leaves, stem, and root to examine the variable anatomical features of *Zantedeschia* species used in this study. The materials are hardened by replacing the water in the materials with paraffin so that they can be cut. The steps required in using paraffin to make a permanent slide [50]. 

The protocol was as follows: Cut a fresh sample and put it in an FAA fixative for 24–48 h, then wash by distilled water and put in different ethanol concentrations (70% and 80%) for 48 h, then transfer sample to 95% ethanol for 2 h, and absolute ethanol for 2 h. After that, transfer the sample to serial concentrations of absolute ethanol and chloroform (3:1, 2:1, 1:1, 1:2 and 1:3) for 2 h for each concentration, then transfer the sample to absolute chloroform for 12 h. Add wax granules to samples three times for three days. (Taste the liquid to be sure that wax replaces chloroform). Discard the wax, and then add a new clean molten wax to the sample to wash it several times. Prepare an L-letter and a glass rod painted with glycerin, pour the molten wax inside the L-letter (remove air bubbles by moving forceps in the molten wax), and then put the sample inside the wax. Pass air from mouth on glass plate surface to dry it, and then put it in water slowly to remove air bubbles.

### 4.7. Cytological Studies 

The geminated beans’ roots were used for chromosomal examination for the calculation of mitotic indices to estimate the chromosomal activities of beans seedlings under different treatments of other plants. Feulgen squash technique was used to stain the chromosomes. The procedure was performed according to the method of Boscaiu et al. [51], as follows:

The root tips were fixed in glacial acetic acid: ethanol (1:3) after a pre-treatment of 2 h in 0.2% colchicine. For staining, the Feulgen standard method was used. After a 5–10 min wash in distilled water, the root tips were hydrolyzed for 30 min in 5 M HCl at room temperature, then transferred to distilled water and stained for 30 min to 1h in leuco-basic fuchsine reagent. Finally, squash preparations were carried out in a drop of 45% glacial acetic acid and examined under the microscope.

The mitotic index can be calculated from a slide with electric microscopy. It is the number of cells containing visible chromosomes divided by the total number of cells in the field of view. Nearly 1000 cells were examined for each different bulb of the species to determine the rate of mitotic division (mitotic index), phase index, mitotic stage index, and to determine abnormalities in chromosomes. Calculations were performed according to the method in Mishra, et al. [52] as follows:

$$M.I=\left(\frac{Number\;of\;dividing\;cells}{total\;number\;of\;dividng\;and\;nondividing\;cells}*100\right)$$

### 4.8. Antimicrobial Activity of Zantedeschia Extract 

The ethanol extract of the two fruits’ peels was tested against Gram-positive bacterial strain *Bacillus cereus*, and Gram-negative bacterial strains *Escherichia coli* and *Pseudomonas aeruginosa*. This assay was applied using the agar plate diffusion method according to Kavanagh [53]. One ml of the standardized bacterial stock suspension (108–109 CFU/mL) was mixed with 100 ml of molten sterile nutrient agar which was maintained at 45 °C. Twenty ml aliquots of the inoculated media were distributed into sterile Petri-dish plates (9 cm). The bacterial cultures were maintained on nutrient agar and 100 µL of plants ethanol extract was inoculated in wells, then incubated at 37 °C overnight. The control bioassay was applied by inoculating only ethanol in wells. 

### 4.9. Statistical Analysis

The gel electrophoresis images were analyzed as 1 (band present) and 0 (band absent). These computations were carried out using Bio-Rad Quantity one (4.6.2) [54]. *Zantedeschia* was aligned with NCBI data to estimate the phylogenetic relationship among these species. In SPSS 21, the data was subjected to an analysis of variance test. Standard deviations, and mean averages were calculated.

## 5. Conclusions

The current study manipulated the floral, digital, molecular, morphological, physiological, cytological, and anatomical identification structures of *Zantedeschia albomoculata*, specifically in the root, stem, and leaf. Descriptive qualitative and quantitative approaches were applied to ensure the enough identification for the plant, especially given that it has few previous records. Morphologically, the leaves are distinctive with the formation of a sheath around the stem forming a spadix. The most characteristic anatomical feature is the formation of raphides and solitary calcium oxalate as a defense mechanism for this plant. The most significant physiological phenomenon is the rapid yellowing and wilting of leaves and a remarkable decrease in chlorophyll content. The essential fatty acids found in these species have multiple applications in many fields. The ethanolic extract has shown antimicrobial activity against some gram-negative and gram-positive bacteria. To sum up, *Zantedeschia albomoculata* is an essential ornamental plant, and it needed to be identified as explained in this work. All these defined parameters enable the plant to be acclimatized and cultivated in the Mediterranean climatic conditions.

## Figures and Tables

**Table 1:** Means of morphological measurements of *Zantedeschia* after four weeks grown on MS in vitro culture.

Morphological Parameters	Means ± SD
Fresh weight (g)	2.573 ± 0.0602
Shoot length (cm)	15.366 ± 1.2503
Branch number	1.666 ± 0.5773
Root number	10.000 ± 2.000
Root length (cm)	13.933 ± 0.3055
Leaf length (cm)	3.600 ± 0.7810
Leaves number	3.000 ± 1.000
Leaf weight (g)	0.3533 ± 0.0351

**Table 2:** Means of physiological parameters after four weeks of *Zantedeschia* grown on MS in vitro culture.

Physiological Parameter	Concentration (mg/g Fresh wt)
Chlorophyll A	0.2135 ± 0.044
Chlorophyll B	0.2844 ± 0.042
Carotenoids	0.6111 ± 0.121
Xanthophyll	1.9328 ± 0.183
Total Protein	56.330 ± 15.25
Total Phenolic Compounds	0.1798 ± 0.024
Total Flavonoids	0.1404 ± 0.0195

**Table 3:** Chemical composition of the essential oil of *Zantedeschia*.

No.	Compound	RT-Min	Area %
1	Benzene, 1-ethyl-3-methyl-	6.266	2.77
2	Benzene, 1,2,4-trimethyl-	6.365	1.09
3	Benzene, 1-ethyl-2-methyl-	6.545	0.50
4	Benzene, 1,2,4-trimethyl-	6.746	2.73
5	Hexadecanoic acid, trimethylsilyl ester	19.645	1.99
6	cis-Vaccenic acid	22.644	31.75
7	Pentadec-7-ene, 7-bromomethyl-	24.734	2.67
8	Glycidyl palmitate	25.943	2.93
9	9,12-Octadecadienoic acid (Z,Z)-, 2,3-dihydroxypropyl ester	29.235	1.20
10	Oleoyl chloride	29.359	6.89
11	Octadecanoic acid, 2,3-dihydroxypropyl ester	30.031	0.66
12	9,12-Octadecadienoyl chloride, (Z,Z)-	30.471	3.65
13	9-Octadecenoic acid (Z)-, oxiranylmethyl ester	30.602	23.75
14	Glycidyl (Z)-9-Heptadecenoate	30.775	1.50
15	Myristic acid glycidyl ester	31.228	1.79
16	Bis(2-ethylhexyl) phthalate	32.510	0.77
17	.beta.-Sitosterol acetate	51.052	1.45
18	.gamma.-Sitosterol	56.048	0.80
19	Pentadecafluorooctanoic acid, dodec-2-en-1-yl ester	59.051	0.84

**Table 4:** The identification of the different compounds in the *Zantedeschia* plant.

No.	Fraction/Compound	Fragment Size m/z
1	Vanillic acid-O-glucoside	152
2	7-Hydroxy-naphthalide	133
3	4-Caffeoylquinic acid	135
4	Methyl-chlorogenic acid	173
5	Dehydroxy-chlorogenic acid	163
6	Roseoside	153
7	Carboxyl-acetyl-rutin	300

## Data Availability

Not applicable.
